# Uncovering the treatable burden of severe aortic stenosis in Australia: current and future projections within an ageing population

**DOI:** 10.1186/s12913-021-06843-0

**Published:** 2021-08-11

**Authors:** Geoff Strange, Gregory M. Scalia, David Playford, Stewart Simon

**Affiliations:** 1 School of Medicine, University of Notre Dame, 32 Mouat St, WA 6160 Freemantle, Australia; 2 Department of Cardiology, Royal Prince Alfred Hospital, Sydney, NSW Australia; 3 Royal Prince Alfred Hospital, Sydney, New South Wales Australia; 4 Heart Research Institute, Sydney, Australia; 5 Prince Charles Hospital, University of Queensland, Brisbane, QLD Australia; 6 Torrens University Australia, Adelaide, SA Australia; 7 University of Glasgow, Glasgow, Scotland

**Keywords:** Aortic stenosis, Aortic valve replacement, Health-services

## Abstract

**Background:**

We aimed to address the paucity of information describing the treatable burden of disease associated with severe aortic stenosis (AS) within Australia’s ageing population.

**Methods:**

A contemporary model of the population prevalence of symptomatic, severe AS and treatment pathways in Europe and North America was applied to the 2019 Australian population aged ≥ 55 years (7 million people) on an age-specific basis. Applying Australian-specific data, these estimates were used to further calculate the total number of associated deaths and incident cases of severe AS per annum.

**Results:**

Based on an overall point prevalence of 1.48 % among those aged ≥ 55 years, we estimate that a minimum of 97,000 Australians are living with severe AS. With a 2-fold increased risk of mortality without undergoing aortic valve replacement (AVR), more than half of these individuals (∼56,000) will die within 5-years. From a clinical management perspective, among those with concurrent symptoms (68.3 %, 66,500 [95 % CI 59,000–74,000] cases) more than half (58.4 %, 38,800 [95 % CI 35,700 − 42,000] cases) would be potentially considered for surgical AVR (SAVR) - comprising 2,400, 5,400 and 31,000 cases assessed as high-, medium- or low peri-operative mortality risk, respectively. A further 17,000/27,700 (41.6 % [95 % CI 11,600 − 22,600]) of such individuals would be potentially considered to a transthoracic AVR (TAVR). During the subsequent 5-year period (2020–2024), each year, we estimate an additional 9,300 Australians aged ≥ 60 years will subsequently develop severe AS (6,300 of whom will experience concurrent symptoms). Of these symptomatic cases, an estimated 3,700 and 1,600 cases/annum, will be potentially suitable for SAVR and TAVR, respectively.

**Conclusions:**

These data suggest there is likely to be a substantive burden of individuals living with severe AS in Australia. Many of these cases may not have been diagnosed and/or received appropriate treatment (based on the evidence-based application of SAVR and TAVR) to reduce their high-risk of subsequent mortality.

**Supplementary Information:**

The online version contains supplementary material available at 10.1186/s12913-021-06843-0.

## Introduction

One of the most common cardiac conditions affecting the progressively aging populations of high-income countries such as Australia is aortic stenosis (AS) [1]. Without timely intervention, severe AS is associated with a very poor prognosis [2]. However, like many other countries, there has been a paucity of reports focusing on the overall prevalence and treatable burden of AS in Australia. A recent AS report from National Echocardiography Database of Australia (NEDA) [3] that assessed the severity of AS and subsequent pattern of survival among 122,809 men and 118,494 women with a mean age of 62 ± 18 years highlighted an urgent need to better understand the burden imposed by this potentially deadly condition. Overall, the indicative prevalence of severe low or high gradient AS among adults being investigated with echocardiography during the overall study period of 2000–2019, was 1.1 and 2.1 % (3.2 % combined), respectively. Actual 5-year mortality ranged from 56 to 67 % in those cases with a native aortic valve and no indication of surgical intervention [3].

Historically, surgical aortic valve replacement (SAVR) has been the preferred intervention for severe AS [4]. However, transcatheter aortic valve replacement (TAVR) has been successfully applied in those with severe AS with high/prohibitive surgical risk [5–7]. Moreover, two head-to-head randomized trials have now reported non-inferiority [8] and superiority [9] comparing TAVR to SAVR in low-risk patients with severe AS, in respect to mortality and subsequent risk of stroke. Consistent with these data, for most of the 6,050 cases within the NEDA cohort who underwent AVR, their post-procedure AV hemodynamic profile and survival outcomes were favourable [10].

Overall, these data suggest more Australians might benefit from AVR. However, without reliable estimates of the treatable burden of AS, this critical number (for health service and resource planning) remains unknown. A series of modelled studies, first published in 2013 [11] and then an updated version in 2018 [12], applied the best available epidemiological and registry data to estimate the following for Europe and North America – 1) the overall proportion of older individuals affected by AS and more specifically severe AS; and 2) proportion of these individuals who had and/or would benefit from a valve replacement procedure (SAVR or TAVR). Given the geographical focus and source data used, these estimates now provide these target regions with (moderate) reliable estimates of the treatable burden of AS. To date, there are no equivalent burden of disease estimates for Australia.

### Study aims

We sought, for the first time, to generate reliable (but inherently conservative) estimates on the prevalence and treatable patient population with severe, symptomatic AS in Australia. Specifically, our aim was to replicate the same robust models recently used to generate contemporary AS-specific projections for Europe and North America [12] as highlighted above with specific modifications relevant to the Australian context. Firstly, this included applying population profiling (denominator) data derived from the Australian Bureau of Statistics (ABS). Secondly, given the Australian context, we aimed to expand our burden of disease estimates to those aged ≥ 55 years (noting the original study applied a single estimate of the overall prevalence of severe AS (numerator) to those aged ≥ 75 years [12]) and by applying age- and sex-specific, incident, and prevalent estimates of AS informed by the recent NEDA Study of AS [3].

## Methods

### Study design

Consistent with previous reports of this type specifically focusing on atrial fibrillation [13] and heart failure [14], we combined official national population data with the best available epidemiological and clinical data to generate estimates of the treatable burden of disease relating to AS. Given the anonymous source and nature of the study data, no ethical approvals were required.

### Study setting

Projections were applied to the entire Australian population and then each State and Territory according to their currently estimated demographic profile. ABS data projecting the future demographic structure and profile were also used to derive future projections on the treatable burden of AS. (https://www.abs.gov.au/Population - Accessed January 2021).

### Study data

The primary analyses presented in this report used age-, sex- and geographic-specific demography data for Australian men and women aged ≥ 55 years for the calendar year 2019. Specifically, the ABS currently provides population projections derived from the official 2016 Census - http://www.abs.gov.au/ausstats/abs@.nsf/mf/3101.0 - Accessed January 2021).

**Fig. 1:**
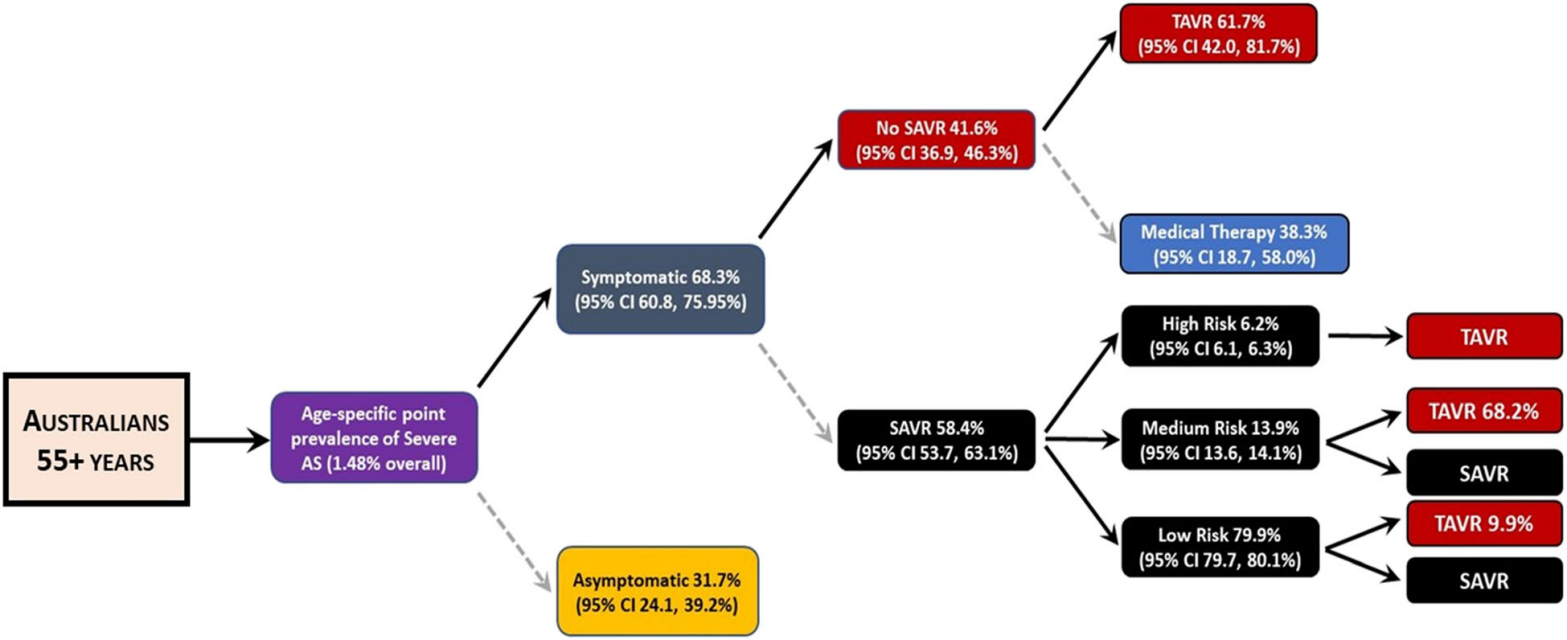
Model/Decision Tree to Determine Treatable Burden of Severe AS in Australia (Adapted from original [12]). Legend: AS = Aortic Stenosis, SAVR = Surgical Aortic Valve Replacement, TAVR = Transcatheter Aortic Valve Replacement, High, Medium and Low Risk based on the Society of Thoracic Surgeons Predicted Risk of Mortality Score of >8%, 4-8% and <4% for SAVR-related mortality, respectively

As indicated above, the primary basis for this report is the recently updated study of the number of treatable and treated patients with severe AS published by Durko and colleagues in 2018 [12]. Specifically, based on an expanded analysis of 37 relevant studies involving 26,402 cases/patients, this study provides a flow-chart (see Fig. 1) of the key estimates/parameters (with 95 % confidence interval, CI) that can be applied (as a primary analysis) to the latest Australian population data (on an age-, sex- and geographic-specific basis) to estimate the pool of treatable severe AS patients per annum. It can also be applied to broad population projections for an increasing pool of older Australians over time; noting that over a decade (from 2006 to 2016) the proportion of Australians aged > 65 years had increased by around 4 % and this proportion (and absolute numbers) will steadily increase.

Specifically, the model developed and subsequently applied in this report provides overall estimates (based on a combination of European and North American cohort studies) on the proportion of patients with severe AS who – 1) are symptomatic and under current expert guidelines, are largely excluded from surgical management; 2) are symptomatic but unsuitable for SAVR but might safely undergo TAVR (the alternative being medical therapy); or 3) are symptomatic and will undergo SAVR; a proportion of whom (particularly due to high-to-medium surgical risk) may benefit from TAVR. The range of derived estimates for each parameter, despite efforts to smooth-out inevitable heterogeneity, are indicative of the methodological weaknesses/biases inherent to source data. It is important to note these treatment pathways are continually evolving as new, lower risk valve interventions are developed and lower the likely threshold of AVR. Without Australian-specific to correct the rates of intervention applied, it is generally acknowledged that intervention rates for severe AS broadly follow those applied in North America.

Where possible, we were able to improve our assumptions and therefore our projections by considering primary profiling and outcome data derived from (NEDA) [3]. This unique study has now captured echocardiographic data on > 750,000 Australians (with no exclusion criteria) being routinely investigated with echocardiography from > 25 centres Australia-wide. With individual data linkage, NEDA also generates real-world, short- to long-term survival data on those affected by common cardiac conditions including AS [3].

### Estimating the prevalence & treatable burden of severe AS

For these analyses, we applied a point prevalence of 3.5 % for severe AS among individuals aged ≥ 75 years, This small adjustment to the original European model [12] reflects recent reports of an increasing incidence of AS in other high-income countries such as the UK [15] and is only slightly higher than that of the NEDA cohort of actively investigated patients [3]. To derive specific prevalence rates for those aged < 75 years, we used the ratio of cases of severe AS observed in each age-band of the NEDA cohort [3]. This resulted in the following age-specific prevalence estimates being applied: ≥75 years, 3.5 %; 70–74 years, 1.2 %; 65–69 years, 0.7 %; 60–64 years, 0.5 %; and 55–59 years, 0.4 %. When applied to the Australian population aged ≥ 55 years (see Supplementary Figure S1), the overall *estimated point prevalence of severe AS is 1.48 %* for 2019; a figure that is broadly consistent with that published and applied previously [16]. The same rates were applied to men and women given that, on an age-specific basis, there were minimum differences based on sex within the NEDA cohort [3].

### Mortality

To understand the potential consequences of no AVR intervention in the setting of severe AS, age- and sex-specific, actual 5-year mortality rates were applied to the estimated case prevalence of severe AS by applying those observed rates within the equivalent patient cohort identified within NEDA who had a native AV throughout follow-up [3]. Although these data only reflect cases referred for echocardiography, they provide both a discussion point and means to validate prevalence estimates when combined with incidence rates (see below).

### Incident cases of severe AS

In the absence of specific population incidence data, we used the differential in prevalence for each 5-year age group (i.e., how many more at risk individuals would develop severe AS over 5-years to reflect the number of prevalent cases in that older age group) to calculate how many individuals would develop severe AS each year. By necessity this means incident cases were only calculated for those aged ≥ 60 years. Applying conservatively derived data from the NEDA cohort [3], the following age-specific, annual incident rates, were applied to the Australian population aged ≥ 60 years for the subsequent (i.e. beyond 2019) 5-year period 2020-24: aged ≥ 75 years, 460 cases; 70–74 years, 40 cases; 65–69 years, 40 cases; and 60–64 years, 20 cases per 100,000 population per annum. When combined, these rates generated an annual incidence of severe AS of 182 cases per 100,000 population per annum within the Australian population aged ≥ 60 years.

### Statistical analyses

All projections are reported as whole numbers and proportions with 95 % CI where appropriate. All statistical analyses are descriptive in nature and population-based; with no inferential statistics applied. Exact estimates are provided in the figures whilst rounded up (to the nearest hundred) figures are provided in text.

## Results

### Prevalent cases of severe AS

**Fig. 2:**
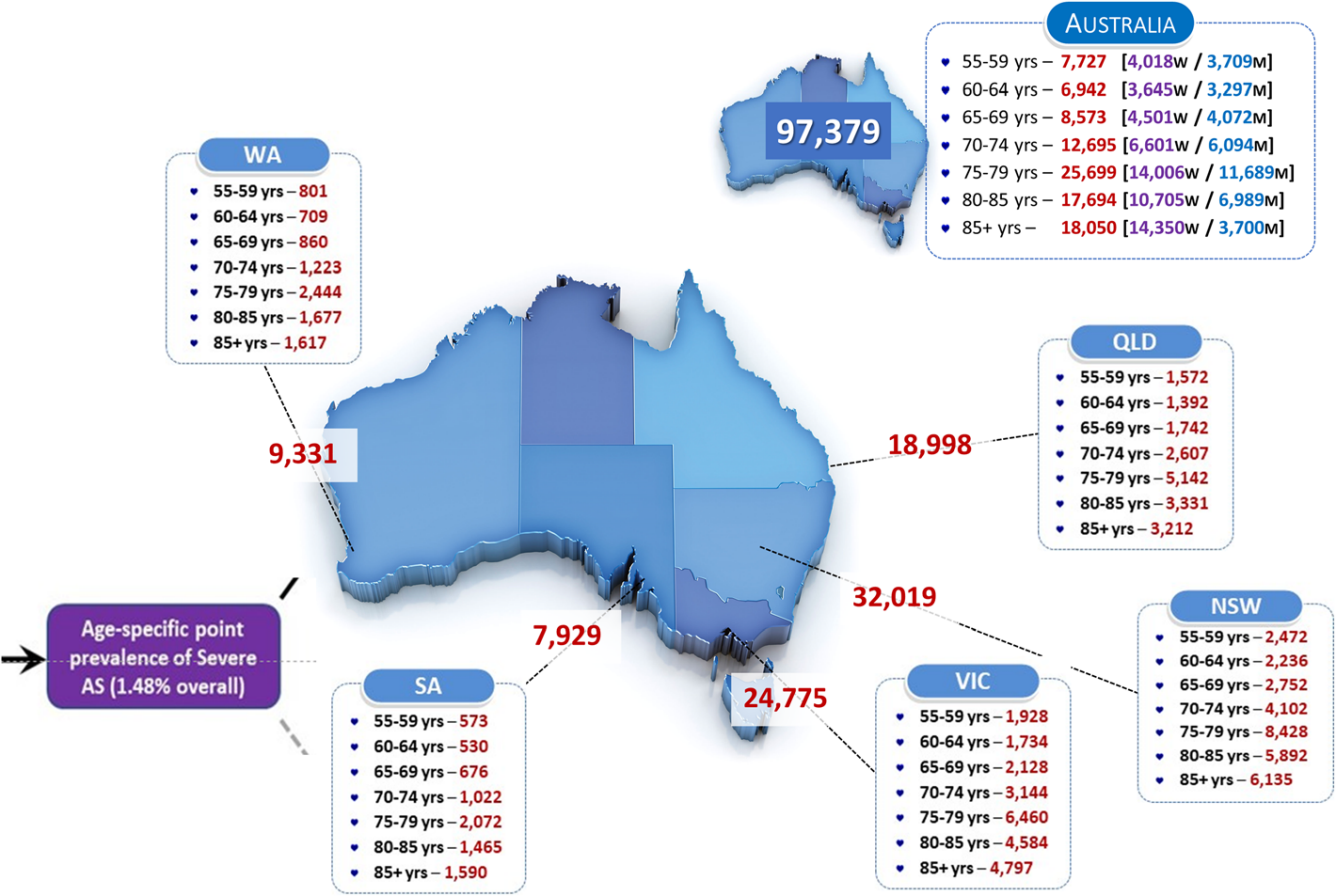
Estimated Point Prevalence and Distribution of Severe AS in Australia

As shown in Fig. 2, we conservatively estimate that around 97,300 Australians are living with severe AS (symptomatic or otherwise). Moreover, assuming just over two-thirds of these cases experience concurrent symptoms linked to the condition, according to contemporary clinical recommendations/ best practice around 66,500 (95 % CI 59,200 to 74,000) people might be considered for an AVR procedure at any one time.

### Aortic valve replacement

**Fig. 3:**
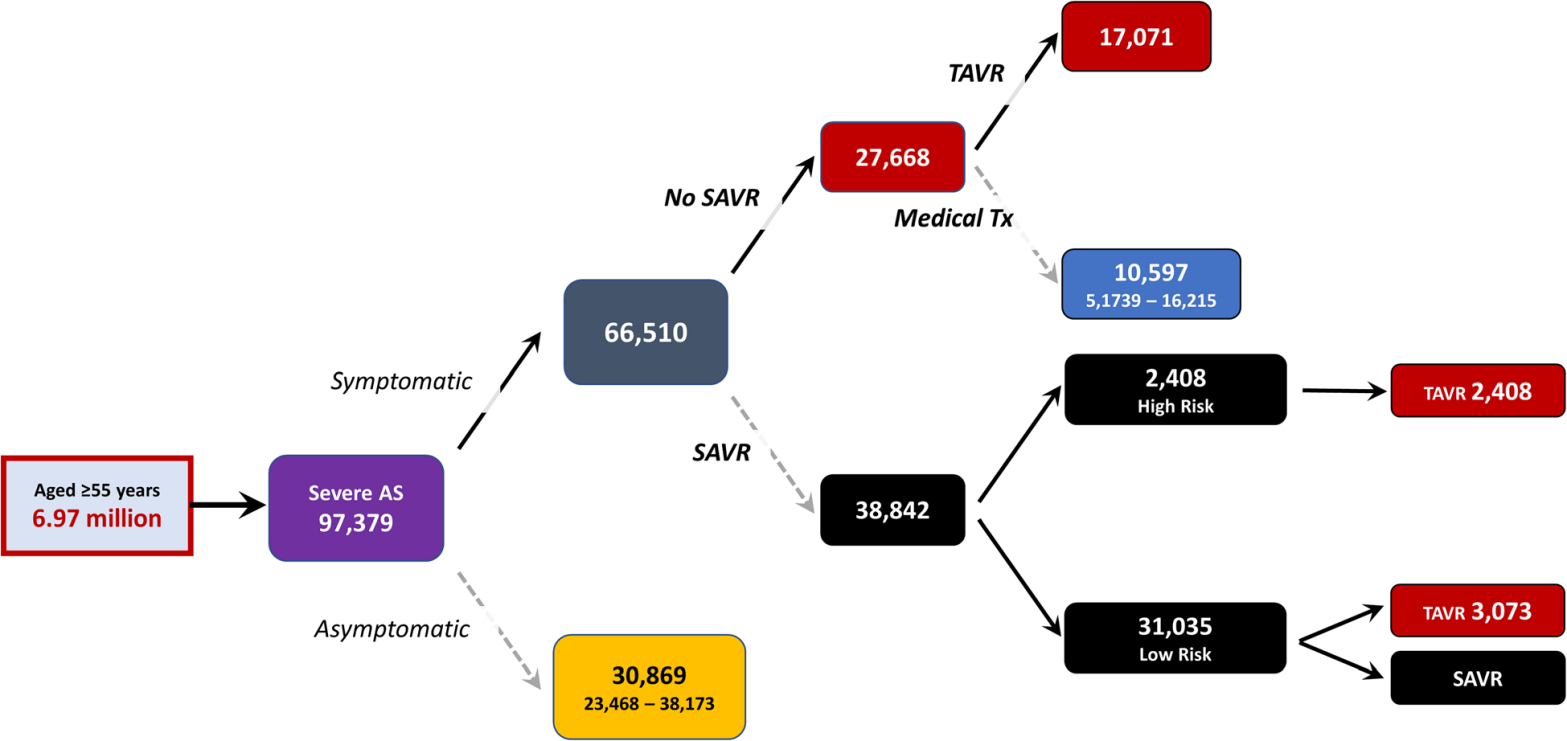
Estimated Treatable Burden/Management of Severe AS in Australia (Based on Prevalent Cases)

Figure 3 summarises the overall treatable burden/management of severe AS when assuming 58.4 % of cases with symptomatic, severe AS would be referred for SAVR (primary replacement or revision) and the remainder (41.6 %) for potential TAVR. On this basis, we estimate that around 38,800 (95 % CI 35,700 to 42,000) Australians aged ≥ 55 years with severe, symptomatic AS might be considered for SAVR; based on the Society of Thoracic Surgeons Predicted Risk of Mortality Score [17]. An additional 17,000 cases (95 % CI 11,600 to 22,600) of the approximately 27,700 people not eligible for SAVR due to high peri-operative risk could be potentially considered for TAVR instead.

### AS-related mortality

**Fig. 4:**
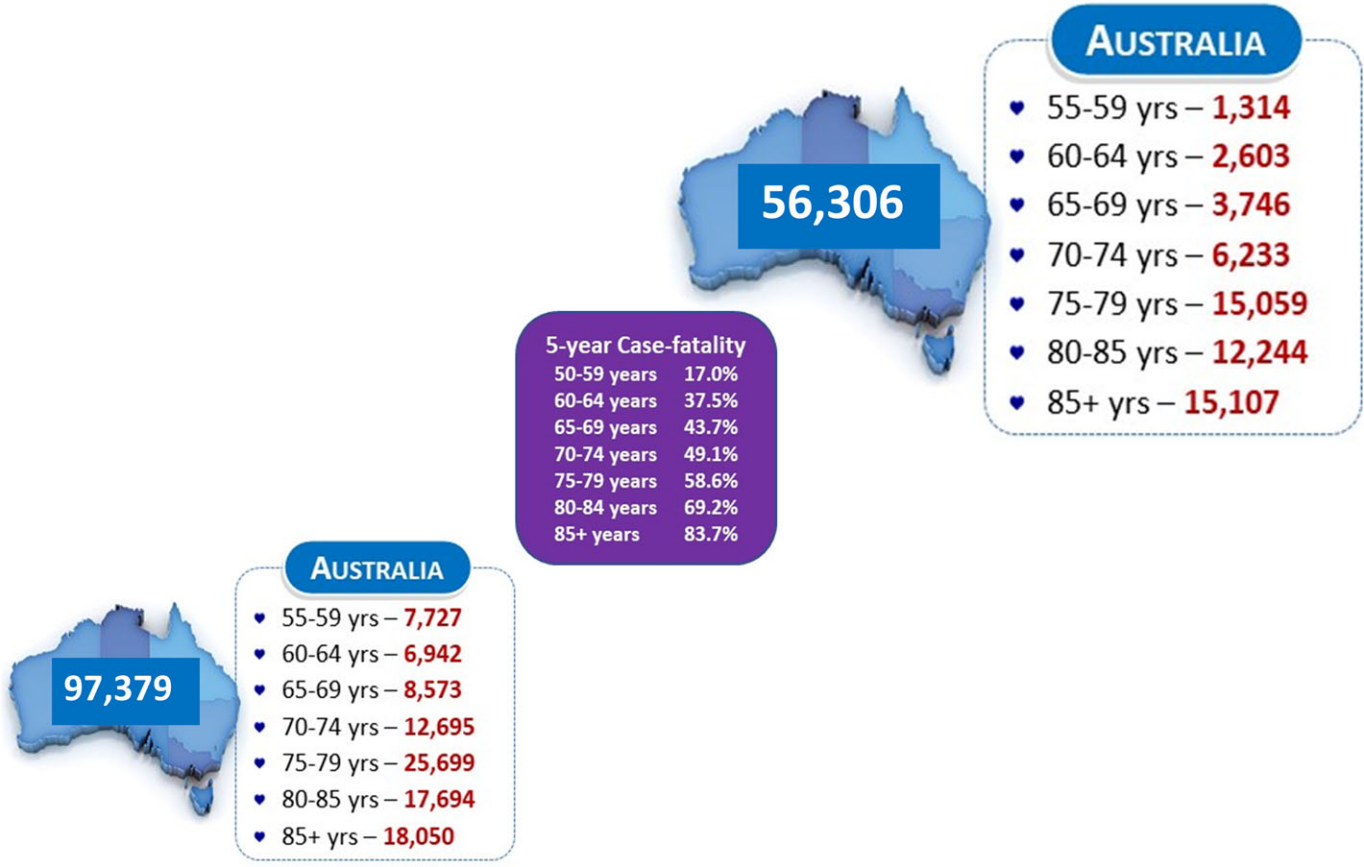
Severe AS Related Mortality

Based on the age- and sex-specific rates of actual mortality (ranging from 17 to 84 % for those aged 55–64 years to ≥ 85 years) observed in the NEDA cohort, we estimate that 56,300 (95 % CI 56,100 to 56,500) of the prevalent population with severe AS of their native valve will subsequently die within the next 5 years - see Fig. 4.

### Incident cases

**Fig. 5:**
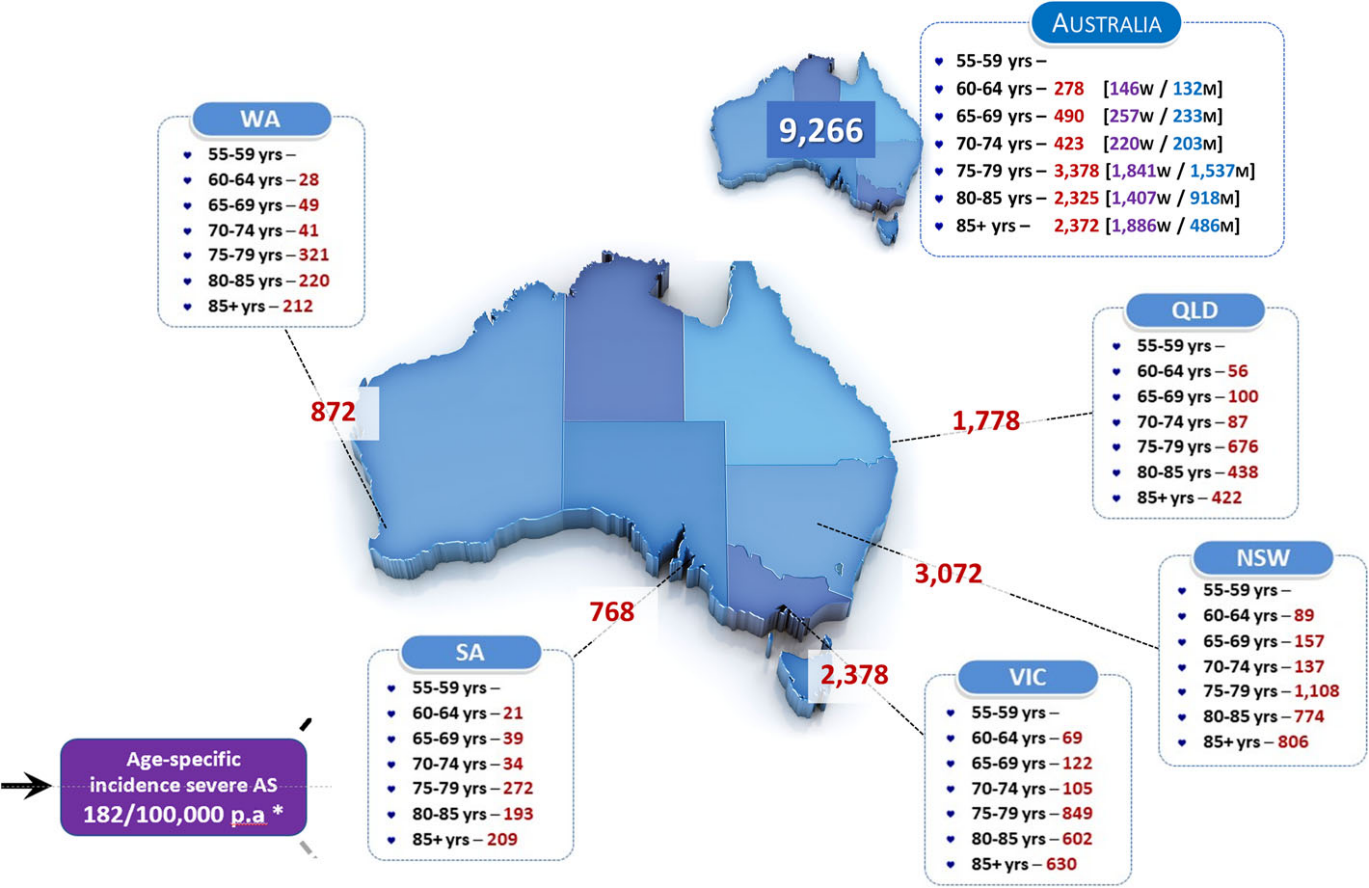
Annual Incident Cases of Severe AS among Australians Aged ≥55 years

As shown in Fig. 5, each year, we further estimate that around 9,300 (95 % CI 9,000 to 9,500) more Australians aged ≥ 60 years will subsequently develop severe AS. Assuming the same pattern of potential AVR procedures (once again based on peri-operative risk status), 3,700 (95 % CI 3,400 to 4,000) and 2,600 (95 % CI 2,300 to 2,900) of the approximate *de novo* 6,300 (95 % CI 5,600 to 7,000) cases with concurrent symptoms linked to the condition, would be potentially managed with/eligible for a SAVR or TAVR procedure, respectively (see Supplementary Figure S2).

## Discussion

In the setting of specific surgical registry reports [18] and recent insightful data generated by the NEDA Study [3] but little else, we present a unique analysis of the potential treatable burden of severe AS in Australia. As with many substantive public health issues that routinely affect a large proportion of the population and is associated with costly treatment and historically poor health outcomes [13, 14], unfortunately there is a paucity of specific burden of disease data to guide health resources and clinical practice. Indeed, worldwide, the natural history and impact of AS remains poorly characterised [19]. This relative lack of data is exacerbated by the rapid progression of disease and high mortality in those affected [20]. It was on this basis we chose to use the best available modelling and projections on the population prevalence and treatable pattern/burden of severe AS [12] and then applied them to the Australian population aged ≥ 55 years with further adjustments/ improvements based on NEDA Study data [3]. Overall, our analyses suggest that close to 100,000 Australians in this at-risk age-group are currently living with this potentially deadly condition. Accordingly, in the next 5 years, more than half of these individuals will die without having undergone an AVR procedure – their risk of dying being two-fold higher on an adjusted basis than their counterparts without severe AS [3]. Without any change in its natural history (there being strong evidence that its prevalence will rise within our increasingly sedentary and obese population [15, 21], this number will likely rise substantially within Australia’s ageing population. At minimum, we estimate that an additional 9,000 Australians aged ≥ 60 years and over will develop severe AS each year.

Overall, based on contemporary management practices in Europe and North America (noting this remains a highly evolving field), we estimated that just under 56,000 prevalent cases would be potentially eligible for a SAVR or TAVR procedure. As also shown by the NEDA Study, successful restoration of AV function with AVR is associated with markedly improved survival [10]. Due to population dynamics (including greater longevity among Australian women) more women than men are likely affected by severe AS, but many of these are aged > 80 years and may have comorbidities that will favour more conservative management options.

Regardless, of the relative accuracy of these projections (noting our critical corrections of the original model [12] based on the very large and robust data derived from the NEDA cohort [3] – see below), these data provide an important context to the largely hidden but substantive burden of disease imposed by AS in Australia. From an individual to societal perspective, it seems clear that due to Australia’s progressively ageing population, a clear strategy to detect and then optimally manage an increasing burden of AS is urgently required. Outcome data derived from close to 350,000 Australians investigated with echocardiography and collectively followed-up > 1 million person-years as part of the NEDA Study [3] further reinforces the need to prioritise AS management. In that study we reaffirmed that severe AS had very poor survival rates (two-thirds dead within 5-years). We also confirmed that when applied, AVR was largely associated with optimal AV hemodynamic profiles and lower mortality [10].

A recent analysis of 18,147 patients (mean age 72 years and 64 % men) with AS who underwent SAVR captured by the Australian and New Zealand Society of Cardiac and Thoracic Surgeons (ANZSCTS) database during 2002–2015, showed that this procedure accounted for 20 % of all adult cardiac surgeries by the end of the study period [18]. In recent years, TAVR has been successfully applied to patients with severe AS and with high/prohibitive surgical risk [5–7]; with two randomized trials reporting the non-inferiority [8] and superiority [9] of TAVR in respect to mortality and subsequent risk of stroke, respectively, when compared to SAVR in low-risk patients with severe AS. A recently reported Australian trial of TAVR applied to 199 intermediate-risk cases of severe AS, is indicative of the changing clinical landscape in this regard [22].

Despite the type of data described above, determining the actual proportion of Australians at risk of poor outcomes associated with AS and then actively treated with SAVR or TAVR, remains problematic; even when reconciling the largely concordant data around the size of the likely active patient population derived from the NEDA Study and those formal projections. However, there does appear to be a disconnect between the number of Australians with severe AS who might benefit either SAVR or TAVR and their subsequent access to these procedures. A recent report from the ANZSCTS database recorded ~ 4,000 SAVR procedures overall in Australia during the period 2009–2015 [18]. Even when accounting for the fact that registry did become truly national until 2015, there appears to be a large shortfall in the expected number of such procedures per annum relative to our projections. This may well be explained by the demographic profile of those undergoing SAVR in Australia. As reported [18], the mean age of SAVR cases is around 72 years of age and only 37 % were female. Alternatively, the mean age of those with severe AS (during a similar timeframe of surveillance) within the NEDA cohort is around 80 years of age and, consistent with this report, more than half of cases were women. Anecdotally, referral for SAVR in the Australian population has typically paralleled North American trends and the presumptions of the modelling (shown in Fig. 1) are based on historical data from a combination of North American and European centres. Given the lag in referral behaviour to new low-risk interventions (e.g., TAVR), by definition, all these assumptions for the modelling will be conservative. Critically, the availability of these new valve technologies has developed rapidly in Australia in the last 5 years and, other than NEDA, there is a paucity of resources to track these changes in real-time.

The recently reported NEDA Study data suggesting that even mild-to-moderate forms of AS are associated with high-levels of mortality approaching that of severe AS within 5-years of follow-up [3], when combined with other contemporary reports [23, 24], are likely to change the landscape of AS management. Specifically, this will likely reflect a recognition that a “watchful wait” approach to determine the transition from moderate to severe AS and also from asymptomatic to symptomatic status [4] may be associated with unacceptably high mortality rates [5]. For example, reflective of concerns around intermediate-to-high risk of surgical mortality (> 4 %) and the evolving efficacy of TAVR versus the more costly and invasive SAVR, in the current analyses, it was estimated that around 9,000 SAVR cases could potentially be replaced with TAVR. However, determining how and where to invest in dedicated screening programs and apply the latest evidence to prolong the lives of those affected in Australia (noting our estimate of approximately 9,000 new cases per annum – of whom > 4,000 would be aged < 75 years) is futile without firm evidence of the number of individuals involved. The noted “disconnect” between the potential and actual number of Australians who derive survival benefits from an AVR, therefore, is likely to increase over time.

### Limitations

As noted, these data do not completely rely on Australian specific data (other than population estimates/demographic structure and NEDA-derived adjustments to original estimates). To partially address this, we have used a conservative estimate of the point prevalence of severe AS and provide 95 % CI for lower and higher and estimates. However, we acknowledge the dynamics of medical management of severe AS (i.e., conservative treatment versus TAVR versus SAVR) in Australia is likely to be different than reported in Europe/North America. The differential clinical uptake and reimbursement of different procedures from a public health to private health perspective within Australia’s increasingly complex and hybrid health care system, is particularly relevant - as is the variable population dynamics of each jurisdiction across the country. Beyond the broader demographic features of Australia and its major jurisdictions, we did not consider other important factors such as socio-economic status and the concentration of particularly high-risk groups (e.g. predominantly younger Indigenous Australians in Central Australia who experience much higher levels of valvular disease and heart failure [25]); nor did we consider the cost-burden of treating severe AS. Applying the growing resources of NEDA, we hope to address most of these issues/limitations in the future, in order to provide greater clarity around the full spectrum of AS in Australia. Finally, it is important to acknowledge that our mortality estimates (even when considering that they are focussed on those with native valves) are discordant with the low rates of mortality reported in trials such as the PARTNER 3 Trial [9]. This discordance only reinforces the benefits of early recognition and expert management of this otherwise deadly condition.

## Conclusions

These unique estimates provide an important insight into the current and future treatable burden of severe AS in Australia. At the most conservative level, the likely number of currently affected Australians aged 55 years and over will soon rise to 100,000 people. Moreover, the number of new cases with this potentially deadly condition is likely approaching 10,000 per annum. Based on current clinical practice/recommendations, around two-thirds of such cases should be actively managed due to their symptomatic status (predominantly with a combination of SAVR and TAVR) and a high-risk of mortality. However, it is unclear if that is truly the case. Whether there is sufficient clinical awareness of AS and pro-active referral patterns (particularly for Australian women) for active management is yet to be determined.

## Supplementary Information

**Additional file 1: Supplementary Figure 1. **Population Distribution of Australians Aged ≥55 years. Legend: Australia is a federated country, comprising the main populated States (Northern Territory and Capital Territory not shown) of Western Australian (WA), South Australia (SA), Victoria (VIC), Tasmania (TAS), New South Wales (NSW) and Queensland (QLD).

**Additional file 2: Supplementary Figure 2.** Estimated Treatable Burden/Management of Severe AS in Australia (Based on Incident Cases per Annum).

## Data Availability

The datasets used are all available on public sources. Australian population data − 1) official 2016 Census (http://www.abs.gov.au/ausstats/abs@.nsf/mf/3101.0); 2) future projection (https://www.abs.gov.au/Population).

## Abbreviations

AS: Aortic stenosis
AV: Aortic valve
SAVR: Surgical aortic valve replacement
TAVR: Transthoracic surgical aortic valve replacement
95 % CI: Confidence interval
NEDA: National Echocardiography Database of Australia
ABS: Australian Bureau of Statistics
ANZSCTS: Australian and New Zealand Society of Cardiac and Thoracic Surgeons
