# Supplementary material for: Uncovering the treatable burden of severe aortic stenosis in Australia: current and future projections within an ageing population
Source: BMC Health Serv Res. 2021 Aug 11;21:790. doi: 10.1186/s12913-021-06843-0 (PMC8356417; doi:10.1186/s12913-021-06843-0)
Supplement: Supplementary file 1 — Additional file 1: Supplementary Figure 1. Population Distribution of Australians Aged ≥55 years. Legend: Australia is a federated country, comprising the main populated States (Northern Territory and Capital Territory not shown) of Western Australian (WA), South Australia (SA), Victoria (VIC), Tasmania (TAS), New South Wales (NSW) and Queensland (QLD). [file 12913_2021_6843_MOESM1_ESM.docx]

**Supplementary Figure 1** Population Distribution of Australians Aged ≥55 years.

**
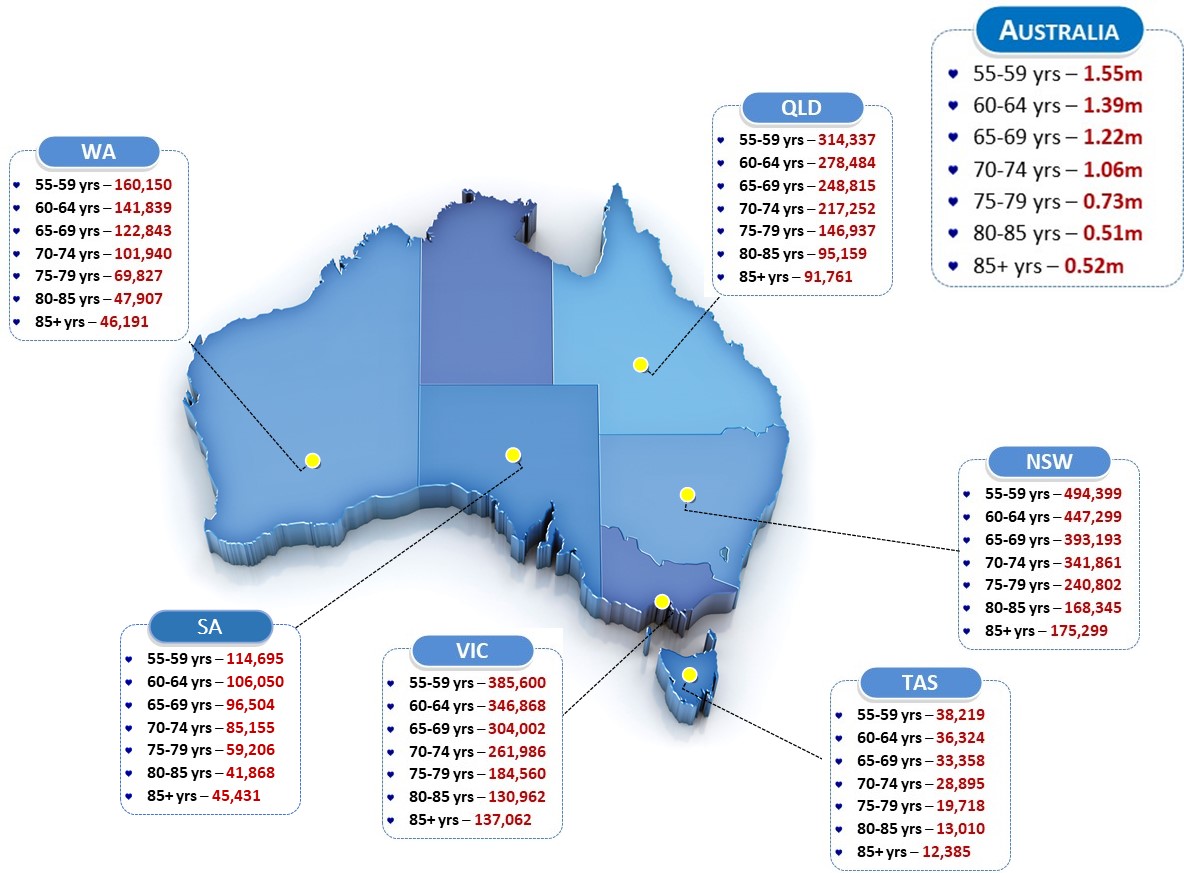
**

***Legend*** *Australia is a federated country, comprising the main populated States (Northern Territory and Capital Territory not shown) of Western Australian (WA), South Australia (SA), Victoria (VIC), Tasmania (TAS), New South Wales (NSW) and Queensland (QLD).*
