# Supplementary material for: Uncovering the treatable burden of severe aortic stenosis in Australia: current and future projections within an ageing population
Source: BMC Health Serv Res. 2021 Aug 11;21:790. doi: 10.1186/s12913-021-06843-0 (PMC8356417; doi:10.1186/s12913-021-06843-0)
Supplement: Supplementary file 2 — Additional file 2: Supplementary Figure 2. Estimated Treatable Burden/Management of Severe AS in Australia (Based on Incident Cases per Annum). [file 12913_2021_6843_MOESM2_ESM.docx]

**Supplementary Figure 2** Estimated Treatable Burden/Management of Severe AS in Australia (Based on Incident Cases per Annum).

**
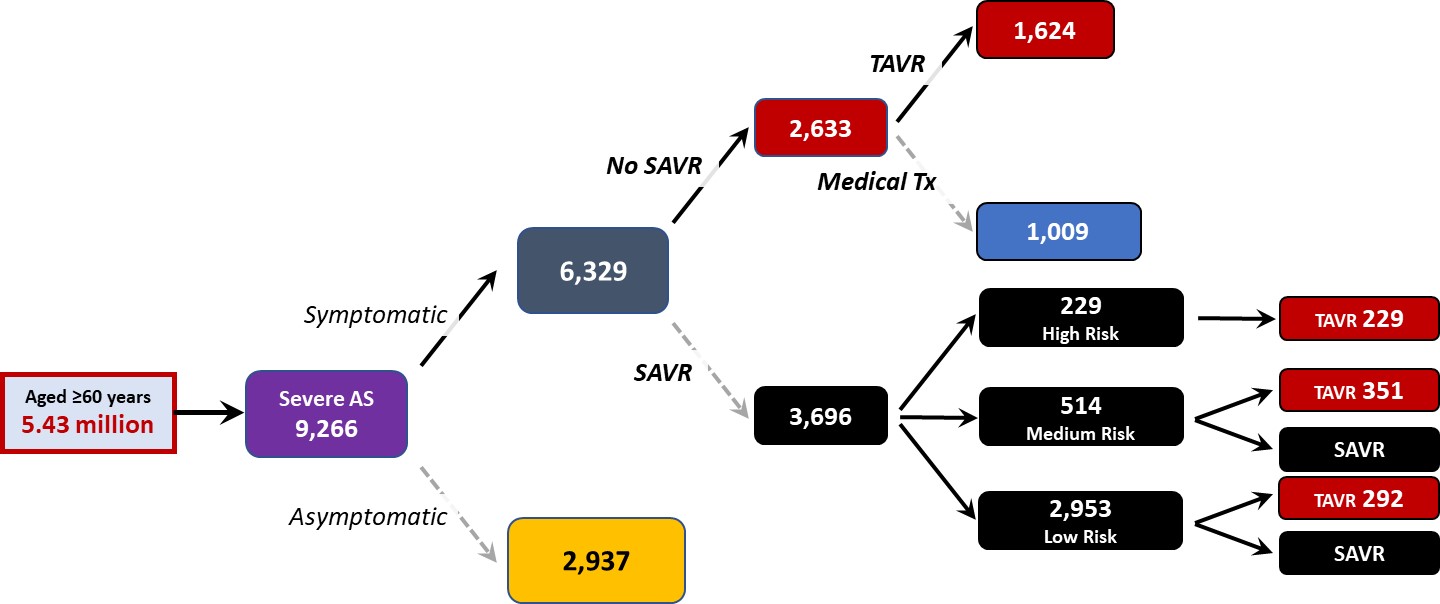
**
